# Editorial: Foliar nutrient analysis in crop species: successes, opportunities and challenges

**DOI:** 10.3389/fpls.2025.1734925

**Published:** 2025-12-08

**Authors:** Theocharis Chatzistathis, Ioannis E. Papadakis, Marco Landi, Patrick H. Brown

**Affiliations:** 1 Institute of Soil and Water Resources, Hellenic Agricultural Organization (ELGO)-DIMITRA, Athens, Greece; 2 Agricultural University of Athens, Athens, Greece; 3 Universita degli Studi di Pisa, Pisa, Italy; 4 University of California, Davis, Davis, CA, United States

**Keywords:** crop nutrition, leaf diagnostics, nutrient use efficiency, precision fertilization, sustainable agriculture

We are pleased to introduce the Research Topic “*Foliar Nutrient Analysis in Crop Species: Successes, Opportunities and Challenges*”. The goal of this Research Topic was to summarize the latest scientific advances in foliar analysis and alternative diagnostic methods to improve nutrient application efficiency, enhance field production, and minimize negative environmental impacts. This Research Topic brings together 9 articles from 71 authors, offering valuable insights into integrated, sustainable, and precision fertilization approaches.

The contributing articles address key themes in modern crop nutrition. Several studies focus on integrated and balanced fertilization, the following:

- Nawaz et al. demonstrated that supplementing standard NP fertilizer with K and Zn in wheat significantly improved physiological performance, nutrient use efficiency, and grain yield, mainly through enhanced chlorophyll content, improved gas exchange parameters, and greater nutrient uptake efficiency.
- Mulugeta et al. found that combining the right carrot variety with an optimal rate of blended NPSB (Nitrogen, Phosphorus, Sulfur, Boron) fertilizer was critical for maximizing vegetative growth and marketable root yield under field conditions in Ethiopia.
- Garg et al. showed that enriched organic formulations, prepared from paddy husk ash and potato peel compost, can serve as effective and sustainable alternatives to farmyard manure, significantly enhancing plant growth, yield, and soil fertility indicators within a multi-crop system.
- Amjadi et al. highlighted that combining complete chemical fertilizer with effective weed control achieved the highest potato tuber yield, while also improving tuber quality traits, such as dry matter and specific gravity, providing practical insights for integrated and sustainable production systems.

Another set of articles explores innovative foliar applications and advanced diagnostics, the following ones:

- Ye et al. found that foliar application of magnesium sulfate effectively corrected Mg deficiency in high-density sweet corn, and significantly increased fresh ear yield and nutrient uptake, while enhancing carbohydrate accumulation and overall physiological efficiency.
- Xu et al. discovered that a low concentration of the fungicide mancozeb not only controlled disease, but also boosted silage maize yield and the relative abundance of beneficial phyllosphere microorganisms, demonstrating that moderate fungicide inputs can modulate the phyllosphere microbiome and improve plant health potential.
- Gill et al. successfully used visible-to-shortwave infrared (VSWIR) spectroscopy to develop strong predictive models for a wide range of macronutrients and micronutrients in winter wheat, demonstrating a powerful, non-destructive method for assessing plant nutrient status that could be integrated into high-throughput phenotyping and digital nutrient monitoring.

Finally, the Research Topic also examines plant resilience and sustainable forage alternatives, via the following articles:

- Wei et al. revealed that nitrate nitrogen enhances drought resilience in *Leymus chinensis* by improving photoprotection efficiency and reducing oxidative stress, through increased non-photochemical quenching and activation of antioxidant enzymes, thereby clarifying how nitrogen form influences drought tolerance mechanisms.
- Pérez-Reverón et al. evaluated twelve native and endemic plant species from the Canary Islands; they found that their nutritional value was comparable to that of traditional forages like alfalfa, emphasizing the potential of native biodiversity to support sustainable livestock feeding systems in arid and insular regions.

All these articles provide a comprehensive overview of the current successes and opportunities in crop nutrition. We hope that this Research Topic will serve as an important resource for improving nutrient management, crop productivity, and fruit quality, while also revealing more efficient and sustainable agricultural practices.

